# Probiotics, Prebiotics and Immunomodulation of Gut Mucosal Defences: Homeostasis and Immunopathology

**DOI:** 10.3390/nu5061869

**Published:** 2013-05-29

**Authors:** Holly Hardy, Jennifer Harris, Eleanor Lyon, Jane Beal, Andrew D. Foey

**Affiliations:** School of Biomedical & Biological Sciences, University of Plymouth, Drake Circus, Plymouth PL4 8AA, UK; E-Mails: holly.hardy@plymouth.ac.uk (H.H.); jennifer.harris@plymouth.ac.uk (J.H.); eleanor.lyon@plymouth.ac.uk (E.L.); jane.beal@plymouth.ac.uk (J.B.)

**Keywords:** probiotics, prebiotics, synbiotics, immunomodulation, tolerance, activation, cytokines, inflammatory bowel disease, cancer, hypersensitivity

## Abstract

Probiotics are beneficial microbes that confer a realistic health benefit on the host, which in combination with prebiotics, (indigestible dietary fibre/carbohydrate), also confer a health benefit on the host via products resulting from anaerobic fermentation. There is a growing body of evidence documenting the immune-modulatory ability of probiotic bacteria, it is therefore reasonable to suggest that this is potentiated via a combination of prebiotics and probiotics as a symbiotic mix. The need for probiotic formulations has been appreciated for the health benefits in “topping up your good bacteria” or indeed in an attempt to normalise the dysbiotic microbiota associated with immunopathology. This review will focus on the immunomodulatory role of probiotics and prebiotics on the cells, molecules and immune responses in the gut mucosae, from epithelial barrier to priming of adaptive responses by antigen presenting cells: immune fate decision—tolerance or activation? Modulation of normal homeostatic mechanisms, coupled with findings from probiotic and prebiotic delivery in pathological studies, will highlight the role for these xenobiotics in dysbiosis associated with immunopathology in the context of inflammatory bowel disease, colorectal cancer and hypersensitivity.

## 1. Introduction

The regular intake of beneficial microorganisms, or probiotics, is an extensively-studied approach for tapping into the health benefits bestowed by commensal microorganisms colonising the gastrointestinal tract (GIT) of the healthy human host. The probiotic strains used for human consumption must be of human origin, non-pathogenic and survive gastrointestinal transit in order to confer health benefits on the host [1]. Owing to their non-pathogenic profile *Lactobacilli* and bifidobacteria are the most commonly used species and significantly influence human health through a range of effects which include; detoxification of xenobiotics [2], biosynthesis of vitamin K [3], metabolic effects of fermentation of indigestible dietary fibre [4], positive influence on transit of luminal contents by peristalsis [5], competition with pathogenic microbes for nutrients and binding sites on mucosal epithelial cells [6] and modulation of the host’s immune response [7].

Non-pathogenic bacteria such as probiotic strains of *Escherichia coli* have been demonstrated to exclude pathogens by suppressing pathogenic growth through the secretion of potent antimicrobial peptides (AMPs) such as the bacteriocin, microsin S [8]. Moreover, co-administration with prebiotics (synbiotics) may work in cooperation to selectively promote the growth and activity of one or more beneficial probiotic species [9,10]. Ingestion of prebiotics alone can stimulate the activity of pre-existing indigenous species which have the potential to be a more cost-effective strategy in positively modifying pre-exisiting commensal microflora [11,12]. Prebiotics are defined as natural or processed ‘functional foods’ which contain biologically active compounds that have documented clinical benefits on health, ranging from prevention of colorectal cancer to modulation of host defences to viral and bacterial infections by altering the interactions between pathogenic and beneficial bacteria [9,13]. The most extensively studied prebiotics are the fructans (inulin, fructo-oligosacharides (FOS)) and galacto-oligosaccharides (GOS) which, owing to their chemical structure, are indigestable in the small intestine and are anaerobically fermented by bacteria in the colon [14,15]. This fermentation of non-digestible dietary fibre/carbohydrate results in the production of short chain fatty acids, (SCFAs—acetate, proprionate, butyrate), that have significant positive impacts on intestinal epithelial cell function, including maintenance of metabolism, proliferation, differentiation and promotion a low pH5 of the gut environment, favouring beneficial microbes with a concomitant reduction in pathogen bacterial growth and viability [16,17]. 

## 2. Commensalism

The human body plays host to communities of beneficial microorganisms whose collective numbers exceed that of human host’s somatic and germ cells [18]. The microbial inhabitants, referred to as the microbiota, mediate key physiological processes in exchange for nutrients and a sheltered habitat in which they are able to reproduce. Strong host selection lead to their co-evolution, whereby indigenous microbes increased host fitness by encouraging cooperation; promoting stable functionality of the gut ecosystem [19]. Metagenomics has revealed the depth of this mutualistic relationship, allowing characterisation of the microbial flora from particular locations of the GIT, regardless of whether the bacteria can be cultured in the laboratory [20]. Although these microbes reside along the length of the gastrointestinal tract, 16s ribosomal sequencing of samples from the colon has identified that the *Firmicutes* and the *Bacteroidetes* are the two dominant phylogenetic types [21]. The human gut microbiome consists of a huge diversity and density of commensal bacteria, which display numerical and strain variation according to anatomical location along the GIT. This species variation is dependent on local environmental conditions and substrate/nutrient availability. Generally, in healthy human hosts, the stomach contains a low density of commensal bacteria with species of *Lactobacillus*, *Streptococcus* and *Helicobacter pylori* predominanting. Bacterial density increases with transit down the GIT, where densities of 10^3^ to 10^6^ cfu/mL are found in the small intestine which facilitate the growth and survival of *Streptococcus* and *Lactobacillus* and finally, *Bacteroides*, *Clostridium*, *Fusobacterium* and *Bifidobacterium* reside in the large intestine/distal gut at densities of 10^8^ to 10^9^ [21]. Thus, due to this strain and density variation of commensal bacteria along the GIT, the consequences to competition with pathogens for binding sites and nutrients, anti-microbial peptide production and even modulation of the host’s immune responsiveness will dramatically vary from one location to another in the gut. Furthermore, these beneficial stable microbiomes, found in the healthy host, are subject to dramatic changes in their resident populations as a consequence of pathological mechanisms: patients with inflammatory bowel disease (IBD) either Crohn’s disease (CD) or ulcerative colitis (UC) exhibit reduced microbial diversity in conjunction with disproportionate quantities of gram-negative bacteria when compared to healthy subjects [22]. 

Exposure to microbes in early life begins with the colonisation of the newborn intestine. Infants born by conventional delivery are colonised at first by vaginal and faecal bacteria of the mother, whereas those born by caesarean section (CS) are initially exposed to bacteria originating from the hospital environment [23,24]. In a study of 165 births, CS infants had lower bacterial counts at one month of age along with significantly decreased populations of bifidobacteria compared to their conventionally born counterparts. Although these differences were no longer apparent at the age of 6 months, CS infants were shown to have greater levels of IgA-, IgG-, and IgM-secreting cells, compared to their conventionally born counterparts, up until the study was completed at 1 year of age [25]. This evidence suggested that lower numbers of initial colonising bacteria, in particular bifidobacteria, may impede establishment of a stable gut microflora during a critical period of immune education and development. This would result in altered collective composition with a lasting influence on immune function far beyond infancy, modifying clearance efficiency of infections and propagation of aberrant immune responses [25]. 

Diet is another important contributing factor to development of the core microbiome during early life. Human milk is a complex biological fluid which supplies fats, proteins, carbohydrates and minerals for the growing infant [26]. In addition to satisfying nutritional requirements, colostrum and milk have been shown to provide early immune education and passive immunity through the synergistic action of many bioactive molecules and cells, including immunoglobulins, lysozyme, lactoferrin [26] as well as being a continuous source of commensals such as *L. acidophilus*, *L. gasseri* [27], *B. bifidium*, and *B. breve* [28]; the compostition of which is mirrored in the infant during the period of breastfeeding [29]. Fernandez *et al.*, proposed that, in addition to bacteria being transferred from mother to infant from the breast skin microbiota, there may be a dendritic cell- or macrophage-mediated trafficking of bacteria from the maternal gut epithelium to the mammary gland epithelium [30]. Moreover, human milk also contains a mix of oligosaccharides which do not nourish the infant but are instead fermented by colonic microbiota in the infant [31]. Human milk oligosaccharides (HMOs) have been shown to selectively promote the growth and activity of bifidobacteria *in vitro* demonstrating prebiotic-like properties [32,33,34]. The exact impact of breastfeeding on the building of the core gut microbiome of the infant is confounded by contemporary practices other than caesarean delivery, including the use of antibiotics and supplementation of nursing with formula milk [35,36]. 

The generation of germ-free (GF) mice has been an essential tool for assessing the impact of composition of the microbiota on the immune system as well as eluding to how particular imbalances can be detrimental to human health. For example, the absence of microbial stimulation in GF animals leads to deficits in lymphoid structures such as the spleen [37] and Peyer’s patches [38] as well as abnormal numbers of immune cell types [39] and expression of cytokine profiles [40]. Specific combinations of species have been shown to drive development of the gut associated lymphoid tissue (GALT) such as *B. fragilis* and *B. subtilis*, thought to enhance microfold cell transcytosis. In particular, protein YqxM (stress response controlled by Spo0A in sporulation of *B. subtilis*) is suggested to play a critical role, although a mutant strain (unable to sporulate but can secrete protein YqxM) was unable to initiate development. Either, *B. subtilis* must be around long enough to induce GALT development or additional sporulation-specific factors are also required [41]. 

The effects of imbalances in gut microflora are not restricted to the gastrointestinal tract and may have an impact on systemic immunity, culminating in allergic disorders such as asthma and atopy [42], and autoimmune disease such as type 1 diabetes [43] and multiple sclerosis [44]. Specific pathogen free (SPF) mice have been shown to have poorly defined splenic tissue architecture and a lower proportion of CD4^+^ T-cells compared to conventional mice, with no difference in CD8^+^ T-cells and CD19^+^ B-cells, an effect attributed to polysaccharide A associated with *B. fragalis* [7]. Such observations further support the ‘hygiene hypothesis’ which proposes that a lack of immune challenges, result in the inadequate maturation of the immune system and predispose individuals to food allergy, asthma [45], IBD, type 1 diabetes and multiple sclerosis [46].

It can be concluded that the establishment and maintenance of a stable gut microbiome, through the appropriate exposure to commensal organisms and their prebiotic substrates as a consequence of birth, breast-feeding, weaning and feeding/infectious challenge, has an important impact on colonisation population dynamics and hence, the well-being of the host. Experimental studies have indicated the importance of these commensal organisms in the development of immune tissues, immune education and protection to immunological insults; dysregulation of these commensal populations resulting in immunopathology. To document the roles played by commensal and probiotic organisms in the host’s immune system, it is imperative to first focus on the site of initial introduction and colonisation, the mucosal epithelial barrier of the gut.

## 3. The Mucosal Epithelial Barrier of the GIT

**Figure 1:**
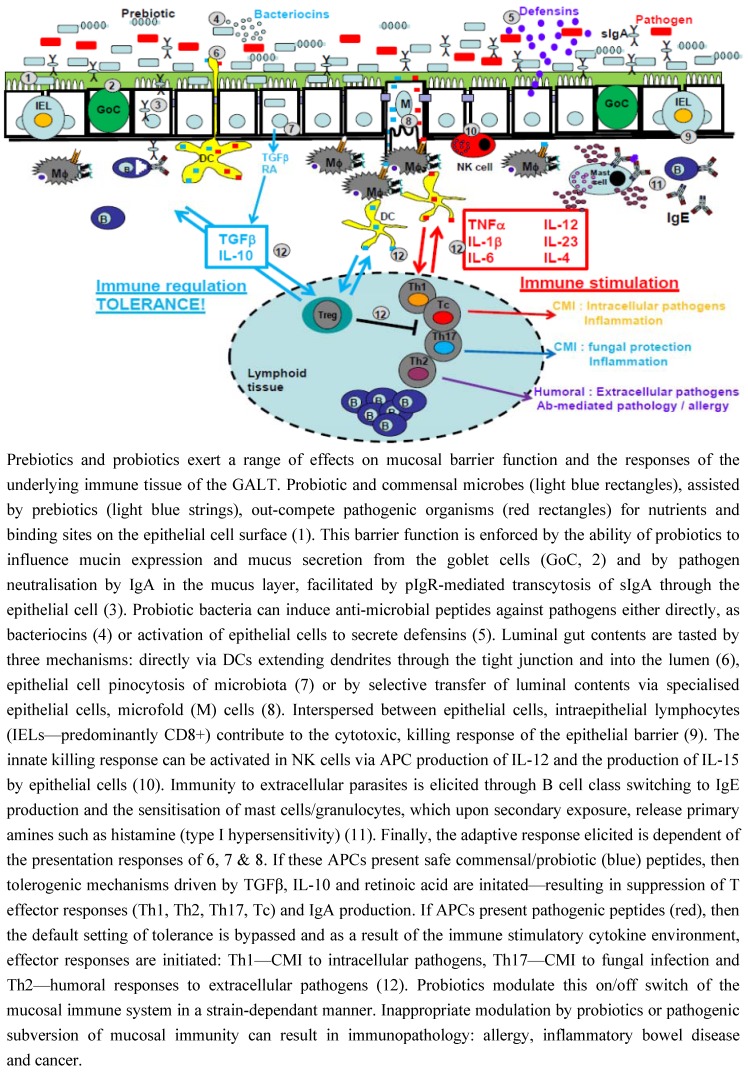
Probiotic and prebiotic modulation of intestinal barrier and immune responses.

The mucosal epithelia of the gut provide an enormous surface area for invading pathogens to gain access to the internal environment of the body. The very characteristics that make the mucosa excellent at physiological activities, such as absorption, also confer vulnerability to pathogenic invasion and infection. The integrity of this barrier can be enhanced by probiotics in a number of ways. Probiotic strains, such as lactobacilli and bifidobacteria, compete for adhesive access to attachment sites on epithelial cells, such as those provided by mannose specific interactions [47], as well as for nutrients which prevents colonisation by pathogenic bacteria [48,49] (Refer to Figure 1). 

### 3.1. Intestinal Epithelial Cells

Barrier integrity is strengthened by commensals and probiotics. Enterocytes express epithelial growth factor receptor (EGF-R), which when activated induces enhancement of the epithelial barrier and tight junctions, probiotic strains have been found to promote this response [50]. Probiotic bacterial cell wall products such as peptidoglycan have been shown to augment apical tightening and sealing of tight junctions by activation of the pattern recognition receptor, TLR-2 [51]. In addition, as yet uncharacterised soluble factors, secreted into conditioned media by *Lactobacillus rhamnosus GG*, have been shown to up-regulate the expression of heat shock protein (hsp) 25 and hsp72 in intestinal epithelial cells *in vitro* [52,53], conferring protection against a variety of cellular stresses including oxidative stress-mediated apoptosis [54,55]. 

### 3.2. Mucus

Integral to gut barrier defence is mucus, composed of mucins. Mucins are a family of heavy molecular weight proteins that display extensive glycosylation and are constitutively secreted by goblet cells interspersed throughout the intestinal epithelium [56]. Mucin polymerisation provides the structural foundation of the mucus, granting protection from pathogens, enzymes, toxins, dehydration and abrasion [57]. *Lactobacillus plantarum* 299v and* Lactobacillus rhamnosus* GG have been shown to up-regulate production of MUC2 and MUC3 intestinal mucins which subvert the adherence of the enteropathogenic bacterium *Escherichia coli* O157:H7 to intestinal epithelial cells, consequently preventing pathogenic bacterial translocation [58]. It is thought that this probiotic-mediated modulation of mucin expression is a strategy for intestinal colonisation of beneficial microbes to the host [59]. Furthermore, mucins may exert prebiotic-type effects as carbohydrate content can account for 90% of their weight [60]. Molecular cloning of glycoside hydrolases from *Bifidobacterium bifidum* have been shown to specifically catalyse oligosaccharides that exist within mucins which can be used as an energy source [61,62]. 

### 3.3. IgA

Protease-resistant IgA is integral to barrier function, playing an important role in trapping pathogens/pathogenic material (neutralisation) in the mucus layer through its ability to bind mucins [63]. Probiotic strains such as *Lactobacillus* GG, *Bifidobacterium lactis* Bb-12 [64] and *Saccharomyces boulardii* [65] have been demonstrated to enhance IgA production and secretion through alteration of the cytokine milieu in the gut mucosa. Probiotic bacteria have been shown to induce epithelial cell expression of TGFβ and IL-10 as well as IL-6 which potentiate IgA production through B-cell maturation and class-switching in favour of IgA [66,67]. Finally, probiotics can induce/augment the expression of polymeric Ig receptors on the basolateral surface of intestinal epithelial cells enhancing transcytosis of IgA through the epithelial cell and into the glycocalyx/gut lumen [68]. 

### 3.4. Antimicrobial Peptides

Also important to barrier defence against pathogenic microbes is the ability of epithelial cells, probiotics and commensals to produce antimicrobial peptides (AMPs). Bacteriocins are antimicrobial peptides produced by the majority of bacterial organisms that are thought to contribute to probiotic functionality by assisting with colonisation [69,70], direct elimination of pathogens [71] and acting as signalling molecules directing other bacteria or the host immune system [72]. Many studies have shown that production of bacteriocins by microbiota can lead to sustained presence of beneficial bacterial strains in the GIT. A study using a *Lactobacillus* strain mix demonstrated an improved clinical outcome of pigs infected with *Salmonella*, attributable to the production of bacteriocins by *Lactobacillus salivarius* [73]. This is supported by a study using *Pediococcus acidilactici* probiotic which reduced the viability of *Listeria monocytogenes* [74]; an effect unobservable *in vivo*. The anti-microbial peptide lacticin also failed to protect against infection from *Listeria monocytogenes* [33], highlighting a possible variance in bacteriocin efficiency *in vivo*. In the case of *C. difficile* infection, a targeted approach using *Bacillus thuringiensis* which produces the narrow-spectrum bacteriocin, thuricin CD, is highly effective at killing *C. difficile* whilst having no significant impact on the microbiota composition [75]. In contrast however, broadspectrum bacteriocins such as lacticin 3147 produced by *Lactococcus lactis* subsp.* lactis* DPC3147 has been shown to negatively impact on members of the *Firmicutes* and *Bacteroidetes* [76]. 

Defensins are a family of highly conserved small cysteine-rich AMPs particularly abundant at mucosal sites where they contribute to the host defence by disrupting the cytoplasmic membrane of susceptible microorganisms [77,78]. Paneth cells, residing within the epithelium at the bottom of intestinal crypts, secrete defensin-rich granules upon exposure to bacterial products such as LPS, LTA and muramyl dipeptide [79]. A study using healthy human subjects demonstrated that probitotic *Escherichia coli* Nissle 1917 was able to induce human beta-defensin (hBD)-2 [80], mediated by flagellin-dependent NF-kappaB- and AP-1 pathways [81]. In addition, probiotic Lactobacilli strains are not only able to up-regulate enterocyte hBD-2 production *in vitro* [82]; some species, such as *Lactobacillus lactis*, have been demonstrated to be resistant to the anti-microbial effects of this defensin [83]. Furthermore, as well as their role as AMPs, hBD1 and hBD2 have been reported to play a more direct role in modulating host immunity, acting as chemoattractants for T cells and immature dendritic cells through binding CCR6 [84].

### 3.5. Intraepithelial Lymphocytes

Invasive enteropathogenic bacteria such as *E. coli*, *Salmonella typhimurium* or *Clostridium difficile* can cause intracellular infection of host cells. Located within the epithelium are intraepithelial lymphocytes (IELs) these are a diverse group of cells, predominantly consisting of CD8^+^ T cells, sub-divided by their differential TCR expression; either the γδ- or αβ-TCR. Intraepithelial γδ cells have been shown to respond to affected enterocytes within hours of infection by secreting the antibacterial lectin, RegIIIγ, or directly lysing cells using a natural killer-like effector killing mechanism. These γδ IELs express the receptor NKG2D which responds to host cells displaying signs of infection and cellular stress [85,86]. Interestingly, a recent study using the TNBS-experimentally induced colitis model demonstrated that treatment with a mix of *L. acidophilus* and *B. longum* probiotics suppressed inflammatory destruction of the gut which was associated with the influx of γδ^+^ IELs, increased CD4^+^ Treg populations and IL-10 within the area and a corresponding down-regulation in CD4^+^ T effector cells and pro-inflammatory cytokines [87].

The Aryl hydrocarbon receptor (AhR) is heavily expressed by IELs, ligation of which is necessary in maintaining IELs within the gut mucosa, preventing their migration away from this site to elsewhere in the system. The ligand for the receptor is found in cruciferous vegetables, and it has been shown that a reduction in AhR expression leads to increased bacterial load in the gut and increased tendency towards colitis [88]. Ligation of the AhR receptor is a mechanism for Treg induction, and has been used experimentally to supress Th2 mediated allergy to peanuts via induction of CD4^+^CD25^+^Foxp3^+^ Tregs [89]. Within the mucosa the AhR signalling pathway was triggered experimentally by *L. plantarum* a common probiotic found in food, and observed to promote inhibitors of the NF-κB pathway which suggested that this probiotic induces tolerance to food antigens [90].

It is thus becoming apparent that the commensal/probiotic, mucus/glycocalyx and epithelial cell barriers are not just physical and chemical barriers to pathogenic infection, but represent a clear communication system resulting in direct modulation of host-driven immune responses.

## 4. Immunomodulatory Role of Probiotic Bacteria

The gut mucosal epithelium not only acts as a barrier to unwanted pathogenic organisms but represents a mechanism of safely and selectively tasting luminal contents of the gut, passing this information underneath the barrier to the immune cells/tissue of the GALT in the lamina propria and beyond in the mesenteric lymph nodes. This selective tasting of the contents of the GIT is the way in which the host tolerates that which is beneficial non-self (through the mechanism of immune tolerance/hyporesponsiveness) and mounts protective immune responses to that which is pathogenic non-self (through humoral and cell-mediated immune mechanisms). The process by which this antigenic information is passed to the underlying cells is crucial to this immune fate: tolerance/suppression *versus* activation. There are generally three mechanisms in which antigenic material is processed and presented to the underlying immune cells and that these mechanisms are controlled by three different types of antigen-presenting cells (APCs) (refer to Figure 1).

### 4.1. Tasting of Luminal Contents

These three main cell types, which communicate information about the microflora and the digesta to underlying immune cells are epithelial cells, specialised epithelial cells called microfold (M) cells and dendritic cells (DCs). Epithelial cells or enterocytes, at the apical surface, are linked by tight junctions preventing the penetration of microbial pathogens; these cells however, can facilitate vesicular bacterial/antigenic transfer across the barrier by receptor-mediated pinocytosis. Once inside the cell, antigenic material can be processed and presented in the context of a major histocompatibility complex molecule (MHC) expressed with co-stimulatory molecules on the basolateral membrane, thereby activating lymphocytes located beneath in the lamina propria [91,92]. Although enterocytes/epithelial cells are antigen presenting cells (APCs); in the presence of inflammation, it has been shown that under normal circumstances they fail to express the co-stimulatory molecules (CD80 or CD86) required for activation of lymphocytes. Thus, they function to induce anergy in CD4^+^ T cells and therefore induce a tolerant environment in the presence of commensals [93]. 

Gut mucosal DCs represent the second mechanism of sampling luminal contents and priming immune activation or tolerisation. In a safe environment of commensal organisms and beneficial dietary components, immune activation is suppressed by TGF-β and TSLP, secreted by epithelial cells in response to commensal bacteria, hence promoting a default non-inflammatory humoral environment [94,95]. Ligation of TLRs on the apical surface of enterocytes has been linked to DC activation which, via a fractalkine receptor CX_3_CR1-dependant mechanism, project arm-like extensions (dendrites) between the tight junctions of the epithelial barrier allowing them to independently sample the luminal contents [96,97]. Upon sampling, DCs become influential APCs, having phagocytosed antigen they can migrate to mesenteric lymph nodes where they stimulate lymphocyte proliferation, or they can activate localised lymphocytic cells. This mechanism is important for the sampling of luminal contents without reducing transepithelial resistance/barrier integrity [98].

The third cell type which enables the cross talk between the microbiota and the host’s immune system is the microfold (M) cell, located within the epithelial monolayer above areas of follicular lymphoid tissue referred to as Peyer’s Patches. Here M cells form a gateway, transcytosing microbes and allowing controlled access to a range of immune cell types, inducing expansion and activation of follicular lymphocytes [99]. Unlike enterocytes they do not secrete anti-microbial components or present antigen, but instead shuttle macromolecules and microorganisms to other effector cells such as DCs and macrophages present in the M cell pocket. However, certain pathogens have evolved to recognise M cells as the entry point into the host’s tissue, thus evading detection by other epithelial surveillance mechanisms [100]. 

### 4.2. Recognition of Pathogenic and Commensal Bacteria

Recognition of luminal bacteria as either commensal or pathogenic is of great importance to the mucosal immune system in eliciting positive immune activatory- or negative, tolerising-responses. Innate pathogen recognition receptors (PRRs) such as Toll-like receptors (TLRs) respond to pathogen associated molecular patterns (PAMPs) and are expressed by enterocytes and mucosal APCs (DCs and macrophages). The binding of PAMPs to these innate receptors triggers intracellular signalling cascades, resulting in the release of specific cytokines, exerting anti-viral, pro- or anti-inflammatory effects on neighbouring cells. The expression of TLRs is down-regulated on the apical membrane of the epithelial barrier in comparison to the basolateral side; TLR2 and TLR4 are expressed at low levels on the apical surface and drive tolerance to LPS and peptidoglycan, expressed in the cell walls of commensal bacteria [101]. Equally, basolateral activation of TLR5 by flagellin, a common component of pathogenic bacteria, leads to a heightened pro-inflammatory response, resulting from the translocation of pathogenic flagellin across the epithelium. No such translocation, hence pro-inflammatory response, was observed for commensal *E. coli* flagellin [102]. Indeed, healthy, homeostatic colonic epithelial cells were observed to be unresponsive to bacterial flagellin whereas flagellin that had gained entry to the basolateral surface elicited a TLR5-dependant inflammatory response [103]. This provides a defensive strategy against virulent pathogens, which gain entry by circumnavigating the antigen processing and presentation pathways. Indeed, exposure to virulent pathogenic bacteria induces epithelial cell secretion of IL-8, a chemokine initiating the recruitment and infiltration of neutrophils and commencement of inflammation [104]. The ingestion of the probiotic culture VSL#3 however, has been linked to a dampening down of this response, reducing IL-8 secretion even in the presence of pathogenic *Salmonella dublin* [105]. 

A large proportion of the indigenous bacteria are Gram-negative which accounts for a high LPS load. Intestinal alkaline phosphatase can be expressed by epithelial cells under the control of LPS, originating from normal microbiota [106]. This enzyme is thought to cleave glutamine and phosphate from LPS moieties [107], leaving a dephosphorylated LPS that is unable to activate TLR4 signalling; effectively suppressing proinflammatory responses such as neutrophil activation [108]. Importantly, the localisation of intestinal alkaline phosphatase is largely confined to the apical surface [109]; allowing modification of luminal LPS whilst allowing an immune response to be initiated upon successful bacterial invasion and breaching of the epithelial barrier. This is indicative of a fine balance of bacterial PAMP recognition that descriminates between activation and tolerisation. Indeed, in addition to positive activatory immune mechanisms, recognition of bacterial PAMPs can exhibit inhibitory mechanisms. These mechanisms include a range of strategies to suppress TLR-mediated activatory signals including; (i) reduction in TLR expression, (ii) expression of shed/secreted receptors (sTLR2, sTLR4, sCD14, sST2), (iii) expression of decoy receptors (SIGIRR, ST2L, RP105) and (iv) expression of endogenous inhibitors of TLR signalling pathways (Myd88s, Tollip, A20, IRAK-M, SARM, TRAIL-R, ATF-3, TRIAD3A and possibly NOD2). These strategies have been described to be employed by a range of cells including IECs and monocyte/macrophage lineage cells that are vital to gut mucosal immune functionality (reviewed in [110]).

### 4.3. Lumenal Contents Determine Immune Fate: Tolerance or Activation?

These processing and presentation pathways mediated by epithelial cells, DCs and M cells are pivotal to immune fate decisions upon tasting the gut luminal contents. In the context of safe non-self or harmful non-self (utilising TLRs), these cells pass on the antigenic information resulting in immune regulation/tolerance or immune activation. In safe, homeostatic environments, antigenic sampling results in mucosal tolerance that is dominated by regulatory T cells (Tregs). CD4^+^ Tregs are key to the negative regulatory component of immune responsiveness, acting to suppress unnecessary inflammation and the differentiation of effector cells, such as T-helper (Th) cells and cytotoxic T-cells (Tc). In comparison to other T-cell subsets, Tregs express increased levels of CD25 (IL-2Rα) and the endogenous co-stimulatory inhibitor, CTLA-4, with the majority of CD4^+^ CD25^+^ Tregs expressing the Treg marker, Foxp3. The presence of Foxp3 is essential for Treg differentiation, leading to the production and secretion of anti-inflammatory/regulatory cytokines, such as IL-10 and TGF-β, which mediate Treg suppressive effects [111,112]. In pathologies whereby negative signalling is defective, the therapeutic reinstatement of a tolerogenic setting, through up-regulation of regulatory mediators or the down-regulation of pro-inflammatory mediators, can result in the resolution of chronically inflamed tissue. One characteristic feature of some probiotics is the ability to suppress pro-inflammatory responses through the up-regulation of tolerogenic mechanisms.

A number of mouse model experiments conducted to observe the effects of probiotic administration have identified a ubiquitous characteristic in both *Lactobacillus* and *Bifidobacterium* strains. Lavasani *et al.*, using mice with developing encephalomyelitis, identified that *L. paracasei* and *L. plantarum* induced CD4^+^ CD25^+^ Foxp3^+^ T-regs in mesenteric lymph nodes leading to increased TGF-β levels and reduced inflammation in the CNS [113]. Other studies have confirmed this immunomodulatory effect, with a range of *Lactobacillus* strains shown to increase TGF-β and IL-10 levels [114,115,116]. Many of the aforementioned studies also found probiotic mediated inhibition of pro-inflammatory cytokines including IFNγ, IL-6 and TNFα, further supporting the role for probiotics in suppression of pro-inflammatory immunity. There is, however, a study which conflicts with these findings, demonstrating the ability of *L. acidophilus* and *L. salivarius* to decrease IL-10 and TGF-β levels in the rectum of BALB/c mice. The study further observed no differences in Treg modulation of bystander T-cell function, between control and probiotic-fed mice [117]. The findings of this study, despite being uncharacteristic, may demonstrate anatomical and strain variance in probiotic immunomodulatory function.

To address the mechanisms behind probiotic induced Treg activation, studies have explored the role of probiotics in modulating DC function. *L. reuteri* and *L. casei* have been found to prime DCs to produce increased levels of IL-10 and inhibit the proliferation of bystander effector T-cells; an effect found to involve probiotic engagement of the C-type lectin, DC-SIGN [115]. *B. breve* has also been identified as a mediator of IL-10 production, however unlike the *Lactobacillus* strains, was found to act through MyD88-dependent TLR-2 signalling in CD103^+^ DCs [116]. Engagement of TLR2 has been shown to result in the rapid release of IL-10, which subsequently inhibits opposing cytokines such as the Th1-polarising cytokine, IL-12 [118] and CD103^+^ DCs are known to induce Foxp3^+^ T-cells in a TGF-β and retinoic acid (RA)-dependent manner [119]. Collectively, this suggests mechanisms by which probiotics interact with DC subtypes to induce a tolerogenic setting predominated by anti-inflammatory cytokines, IL-10 and TGF-β. These studies also highlight *Lactobacillus* as activators of conventional DCs and *Bifidobacterium* as activators of CD103^+^ DCs, implying strain variance in DC-subset targeting and functionality. 

### 4.4. Cytokines Are Pivotal to This Immune Cell Fate

It is well established that immune fate decisions (activation or tolerance) are made by immune cells, which are activated by, and elicit an effector response by specific functionality and profiles of the immune cell signals, cytokines. Environmental cytokines can elicit pro-inflammatory responses (TNFα, IL-1β, IL-6, IL-8, IL-15) and anti-inflammatory/suppressive responses (IL-10, TGFβ), through the direction of a wide array of effector cells which include granulocytes, macrophages, DCs, T & B cells. In addition, cytokines drive Th1 differentiation (IL-12) hence CMI via IFNγ, Th2 differentiation (IL-4) and humoral responses via IL4, IL-5, IL-13, IL-10; Th17 differentiation (TGFβ, IL-1β, IL-6 & IL-23) and anti-pathogen responses via IL-17A and IL-22, Treg differentiation (IL-10, TGFβ, IL-35) and suppression via the production of IL-10, TGFβ and IL-35 (refer to Figure 1). There is a wealth of research literature which documents the ability of probiotics to modulate cytokine production; either via immune activation/augmentation, immune deviation or suppression. Modulation of such cytokine expression will have an appreciable impact on immune functionality and represents a clear avenue of manipulation for probiotic use in the treatment and prophylaxis of immunopathology. Many Lactobacillus strains have been described to induce IFNγ and IL-12, which are Th1-cytokines associated with CMI and NK activity whereas other Lactobacillus strains both augment and suppress the Th2-associated cytokines, IL-4 and IL-5, which drive humoral immune responses. More recently, Evrard *et al.* [120], has described *L. rhamnosus* to induce IL-23, an IL-12 family member associated with Th17 differentiation and pro-inflammatory responses. Additionally, a wide range of both bifidobacteria and *Lactobacillus* induce expression of the anti-inflammatory/regulatory cytokines, TGFβ and IL-10, associated with Treg suppressive function/tolerance (for a full citation of probiotic modulation of T cell differentiation/functionality, refer to Table 1).

**Table 1:** Probiotic strains differentially modulate T cell differentiation and effector cytokines.

Cytokines (Immune Response)	Cell system	Response	Probiotic strain	References
IFN-γ & IL-12 (Th_1_-associated, CMI and NK cell activity)	PBMCs	Increase	*L. rhamnosus*	[121]
			*L. plantarum* *L. lactis* *L. casei* *L. rhamnosus* GG	[122]
			*L. lactis* W58	[123]
			*L. casei* Shirota	[124]
			*L. casei* Shirota	[125]
			*L. paracasei* *L. salivarius*	[126]
			*B. longum* W11	[127]
			*L. rhamnosus* *L. gasseri* *B. bifidum* *E. coli* (TG1)	[128]
			*L. casei* Shirota	[129]
			*L. plantarum* strains	[130]
	PBMC-Mo	Increase	*S. aureus* *L. johnsonii*	[114]
	PBMC-DCs	Increase	*L. salivarius* *L. rhamnosus* Lcr35	[131] [120]
	PBMC-NK cells	Increase	*L. acidophilus* *L. reuteri*	[132]
	Myeloid DCs	Increase	*L. gasseri* *L. johnsonii* *L. reuteri*	[133]
	PBMC-NK cells	Decrease	*B. bifidum*	[132]
IL-23 & IL-17 (Th_17_-associated, pro-inflammatory)	Mo-DCs	Increase	*L. rhamnosus* Lcr35	[120]
	PBMCs	Decrease	*B. breve* LGG	[134]
	Caco-2 cell line	Decrease	*L. plantarum*	[135]
IL-4 & IL-5 (Th_2_-associated, humoral)	PBMCs	Decrease	*L. plantarum* *L. lactis* *L. casei* *L. rhamnosus* GG	[122]
			Bifidobacteria	[123]
			*L. rhamnosus* *L. gasseri* *B. bifidum*	[128]
TGF-β (Treg-associated, anti-inflammatory)	PBMCs	Increase	*B. longum*	[136]
	Epithelial cells	Increase	*B. lactis* *L. johnsonii*	[137]

Overview of studies documenting the probiotic strain-specific effects on Th1 cytokines (IFNγ and IL-12) associated with cell-mediated immunity, Th17 cytokines (IL-23 and IL-17) associated with pro-inflammatory anti-pathogen responses, Th2 cytokines (IL-4 and IL-5)—humoral immunity and Treg (TGFβ) associated with immune tolerisation/suppression. All studies are human studies utilising a range of cell sources: peripheral blood mononuclear cells (PBMCs), NK cells, DCs, monocytes (Mo) and Caco-2 gut epithelial cells.

In addition to differentiating CD4^+^ T helper cells to distinct lineages responsible for cell mediated immunity and humoral immune responses, probiotics also have an important role in the modulation of innate inflammatory responses important for early, non-specific anti-pathogen responses and a potential role in the regulation of chronic inflammatory responses. A range of both *Lactobacillus* and *Bifidobacterium* species augment the secretion of TNFα, IL-1β and IL-6 by PBMCs, DCs, monocytes, macrophages and epithelial cells. In contrast there is both a differential and overlapping probiotic strain induction of the anti-inflammatory cytokine, IL-10. Of particular interest is the suppressive effect of *L. casei* Shirota on PBMC production of IL-10 [129], an effect which may be explained by the documented effect of LcS on the induction of the Th1-polarising cytokine, IL-12. (For a full citation of probiotic modulation of inflammatory cytokines, refer to Table 2).

Finally, one of the greatest producers of cytokine is the tissue macrophage. These cells are present in large numbers in the lamina propria of the GIT and, as such, play an important role in driving immune responsiveness in the gut. These mucosal macrophages exhibit a degree of functional plasticity which is determined by the local tissue environment. As such, macrophages can exist as M1-like pro-inflammatory and M2-like anti-inflammatory/regulatory subsets (reviewed in [138]). Recently, probiotic strains have been described to differentially regulate macrophage cytokine production in a strain- and subset-specific manner [139,140]. The inflammatory response being dictated by both the probiotic strain and which macrophage subset is being activated, hence macrophage populations can display differing inflammatory outcomes as a consequence of which subset is predominant in the tissue environment being challenged. Of relevance to prebiotic research, the SCFA, butyrate, produced as a consequence of anaerobic fermentation of prebiotic non-digestible carbohydrates, has also been demonstrated to play a role in macrophage cytokine production; again, the inflammatory cytokine outcome being determined by macrophage subset [141]. Thus, cytokines determine immune responsiveness to commensals, pathogens or in the case of dysregulation, immunopathology.

**Table 2:** Probiotic strains differentially modulate pro- and anti-inflammatory cytokines.

Cytokines (Immune Response)	Cell system	Response	Probiotic strain	References
TNF-α and IL-1β (Pro-inflammatory)	PBMCs	Increase	*L. rhamnosus* *L. bulgaricus* *S. pyogenes*	[121]
			Bifidobacteria	[142]
			*L. casei* Shirota	[124] [129]
			*L. salivarius* *L. fermentum*	[143]
			*L. plantarum* strains	[130]
	PBMC-DCs	Increase	*L. rhamnosus* Lcr35	[120]
	Myeloid DCs	Increase	*L. reuteri*	[133]
	Epithelial cells	Increase	*L. sakei*	[137]
	Macrophage subset cell line	Increase and decrease (subset-specific)	*L. casei Shirota*	[139] [140]
	THP-1 cell line	Decrease	*L. reuteri*	[144]
IL-6 (Pro-inflammatory)	PBMCs	Increase	*L.rhamnosus* *L. bulgaricus* *S. pyogenes*	[121]
	Epithelial cells	Increase	*B. lactis* Bb12 *L. casei* CRL431 *L. helveticus* R389	[145] [146]
	PBMCs	Decrease	*L. casei* Shirota	[129]
IL-10 (Anti-inflammatory)	PBMCs	Increase	Bifidobacteria DNA	[147] [123]
			Bifidobacteria	[142]
			*B. longum* W11	[127]
			*L. fermentum*	[143]
			*L. acidophilus* *L. plantarum* strains	[130]
			*L. acidophilus* *L. reuteri*	[132]
	PBMC-NK cells	Increase	*B. bifidum* VSL#3 *L. reuteri*	[147] [115]
	Blood-DCs	Increase	*L. plantarum*	
	Mo-DCs	Increase	*L. casei* *L. rhamnosus* Bifidobacteria	[148] [149]
	Mo-DCs	Increase	*B. infantis*	[150]
	Mo-DCs, mDCs, pDCs	Increase		
	PBMCs	Decrease	*L. casei* Shirota	[129]

Overview of studies documenting the probiotic strain-specific effects on the pro-inflammatory cytokines (TNFα, IL-1β and IL-6) and the anti-inflammatory cytokine, IL-10. All studies are human studies utilising a range of cell sources: peripheral blood mononuclear cells (PBMCs), NK cells, DCs, THP-1 pro-monocytic cell line, macrophage subsets and intestinal epithelial cells.

## 5. Immunopathology and Probiotic/Prebiotic Immunomodulation

### 5.1. Th1/Th17-Dominant Pathology, Crohn’s Disease

The balance between humoral and cell mediated immunity is important for a healthy immune response, there are a range of pathologies in which a bias in the cytokine and cell differentiation profile are observed. In these cases a dysbiosis of the gut microbiota is often seen, thus the use of probiotics to counteract this has been the focus of many research papers. The understanding of individual variations in gut flora is widening with the recent advances in DNA sequencing and proteomics technologies, allowing in-depth analysis of the strains present in the human GIT, both in health and disease. There is however often a genetic predisposition which leads to an altered response to bacteria, here we review the mechanisms underlying pathology and the potential for using probiotics and symbiotics as a therapeutic tool. Crohn’s is an inflammatory bowel disease, which can affect any part of the GIT; it is characterised by transmural granulomatous inflammation with high expression of IL-12/IL-23 and an associated predominance of CD4^+^ Th1/Th17 cells, leading to the secretion of IFN-γ, TNFα and IL-17 [151] (refer to Figure 1).

Th17 cells are a CD4^+^ expressing Th1-like subset, activated by IL-23 and IL-6 to produce IL-17, IL-22 and IL-26 (reviewed in [152]). Pro-inflammatory, Th17s and IL-17 are of growing importance to immunological research due to their emerging role in inflammatory pathologies including rheumatoid arthritis, Crohn’s disease, cancer and dermatitis [153]. As probiotics are able to modulate both Th1 and Th2 mediated responses, attention drew to potential use in modulating Th17 cells. Several studies have focussed on probiotic modulation of IL-17 and IL-23. Paolillo *et al.* [135] found *L. plantarum* treatment with LPS-activated Caco-2 epithelial cells reduced IL-23, suggested to be a TLR-2 dependent mechanism; a cytokine finding supported by Ghadimi *et al.* [134] who observed a reduction in IL-17 and IL-23 in PBMCs co-cultured with human intestinal cells and treated with *B. breve* and *L. rhamnosus* GG. In contrast, a study using human monocyte-derived DCs found IL-23 to be induced upon treatment with *L. rhamnosus* [120]. Evrard *et al.* further found *L. rhamnosus* to increase CD86 and DC-SIGN expression on human DCs suggesting the effects of Lactobacillus on Th17 activation to be mediated through modulation of DC function [120]. These co-culture system studies are more applicable to *in vivo* settings; in which case particular probiotic strains may have a role in inducing Th17-mediated immunity through modulation of DCs affecting down-stream pro-inflammatory cytokine expression.

Crohn’s sufferers display a shift in commensal bacterial populations towards higher numbers of Gram-negative *Proteobacteria* and lower numbers of Gram positive *Firmicutes*, there is also a dysbiosis in the genera *Bacteroides* with a higher expression of *B. ovatus* and *B. vugatus* and lower expression of *B. uniformis* [154,155]. It is believed that a dysregulated pro-inflammatory response is elicited in those with genetic mutations in pathogen-sensing receptors such as the *CARD15* gene encoding NOD2, a cytosolic protein expressed by epithelial cells, paneth cells, dendritic cells and macrophages and is involved in the sensing of bacterial cell wall peptidoglycan [156,157]. The NOD2 pathway is linked to activation and regulation, of NF-κB and expression of proinflammatory cytokines TNFα IL-1β, IL-12 and anti-bacterial peptides, as well as transcription of apoptotic genes. There is some disagreement however, as to the impact mutations may have in Crohn’s disease, as there are 30 function mutations in NOD2 thus the impact on immunity is highly variable [156,158]. It would seem that an increase in IL-1β may be partially responsible [156]. Although it has been suggested that NOD2 plays a role in the production of anti-inflammatory IL-10 and TGF-β and thus loss of function mutations may result in a loss of tolerance to commensals [159]. It is believed that these mutations, acting in combination with the strains of bacteria present in the GIT, result in an excessive proinflammatory response [158]. As previously noted there is a marked reduction in the numbers of *Firmicutes* found in the GIT of patients with Crohn’s, specifically the beneficial commensal *Faecalibacterium prausnitzii* [160]. A recent study used this strain as a probiotic both *in vivo* and *in vitro*, and found that oral administration of the live bacterium lead to reduced evidence of experimental colitis in mice [160]. A marked increase in IL-10 secretion and significant reduction in IFNγ and IL-12 production was seen in PBMC exposed to this probiotic, thus it is suggested as a potential therapeutic strategy in Crohn’s disease [160,161]. Interestingly, there could be a correlation between this bacterium and transepithelial resistance as *F. prausnitzii* is a butyrate producing bacteria, and butyrate is a metabolic source for the catabolism of ATP vital for host epithelial cell metabolism [161,162]. A diet rich in prebiotic short chain fatty acids provides a supporting role for the butyrate-producing commensals [163,164]. The dietary prebiotic inulin which is contained in bananas, tomatoes, onions, garlic and Jerusalem artichokes has been shown to be a prime source of nutrients for *F. prausnitzii* and therefore future developments in the treatment of Crohn’s could involve symbiotic preparations of inulin and *F. prausnitzii* [165,166]. (Refer to Table 3).

**Table 3:** Probiotic strains, prebiotics and synbiotics differentially modulate immunopathology.

Pathology	Response	Probiotic/Prebiotic	References
Crohn’s	↓ IFN-γ, IL-12	*F. prausnitzii*	[160]
	↑ IL-10	Fructo-oligasaccharides	[167]
Ulcerative colitis	↓ IL-1β, TNF-α, IFN-γ, IL-12	*L. plantarum* 299v	[168]
		LGG	[169]
	↑ IL-10	LGG	[169]
	↓ β-defensins, TNF-α, IL-1, CRP	*B. longum/*Synergy 1	[170]
	↓ CRP	*B. longum/*psyllium	[171]
	↓ adherence of B. vulgatas	LGG	[172]
	↓ expression of tight junction proteins	VSL#3	[172]
	↓ tissue inflammation	VSL#3	[173]
	↑ no. γδ IEL ↓ no. γδ T-cells in lamina propria ↑ no. T-regs	*L. acidophilus* + *B. longum*	[87]
	Reshape microbiota composition	VSL#3	[174]
Colorectal Cancer	↓ aberrant crypt formation ↓ cecal pH	Bifidobacteria+ *Lactobacilli* + Inulin+ Oligofructose	[175]
	↑ SCFA production ↓ IL-2 and iNOS	Bifidobacteria+ *Lactobacilli* + Inulin + Oligofructose	[176]
Colorectal Cancer	↓ H_2_O_2_ ↓ tissue inflammation ↑ catalase activity	*L. lactis*	[177]
	↓ IL-2 and iNOS	*B. lactis*	[176]
		*L. rhamnosus*	[178]
	↑ angiostatin ↑ no. CD4+ T-cells ↑ IL-17 and TNF-α	VSL#3	[179]
	↓ CXCR4 mRNA expression ↓ MHC-class 1 ↓ tumour growth ↑ CT-26 cancer cell apoptosis	*L. acidophilus*	[180]
Allergy	↓ IL-4 and IL-5	*L. plantarum*	[122]
	↑ IL-12 and IFN-γ	*L. lactis*	[181]
		*L. plantarum*	[128]
	↓ IL-5, IL-4 and IL-13	*B. breve* + oligosaccharide	[182]
	↑ no. CD4^+^CD25^+^Foxp3^+^ T-regs	AhR ligand (TCDD)	[89]
	↓ IL-12 ↔ no. T-regs	*B. breve* + oligosaccharide	[183]
	↑ no. bifidobacteria colonies ↓ no. *Clostridium* colonies ↓ faecal pH ↔ IgE ↔ levels of acetic acid	*B. breve* + oligosaccharide	[184]
Coeliac	↑ no. CD4+ T-cells ↑ IFN-γ	*L. casei*	[185]
	↑ transepithelial resistance	*B. lactis*	[186]
	↑ IL-12 and IFN-γ	Shigella CBD8	[187]
	↑ TNF-α	*B. bifidum* *L. paracasei* *L. fermentum*	[187] [188]

Overview of studies documenting the role of probiotic strains, prebiotics and synbiotic formulations and their effects on Crohn’s disease, Ulcerative colitis, colorectal cancer, allergy and coeliac’s disease. All studies are human studies detailing potential modulation of a variety of disease markers and immunological functional readouts. Arrows indicate decreases (↓), increases (↑) or no change (↔).

In addition to Th1 cells, Th17 cells are also prevalent in this pathology due to an imbalance in the suppressive Treg population and proinflammatory Th17 cells, with a bias towards the latter. This effectively tips the balance away from tolerogenic Treg activity towards chronic inflammation due to increases in Th1/Th17 and IL-12/23 expression [189,190]. A recent clinical trial used prebiotic fructo-oligosaccharides in Crohn’s disease patients and found an increase in the numbers of immune-regulatory dendritic cells and heightened the concentration of IL-10 secreted by intestinal dendritic cells, yet these factors did not reduce the clinical presentation of the disease [167]. A further trial, using a symbiotic combination of *Bifidobacterium longum*, *B*. *breve* and *Lactobacillus casei* with prebiotic psyllium, which comes from barley however, resulted in a significant decrease in clinical scoring of the pathology [191]. Just what the beneficial effects of these symbiotic formulations are to the immunological mechanisms will be a focus of future research endeavours. It can be suggested however, that any beneficial effect would result from immunomodulation of DC/APC function, cytokine production and T-helper differentiation/effector function towards a regulatory/suppressive phenotype.

### 5.2. Immunopathology: Th2-Dominant Pathology, Ulcerative Colitis

Ulcerative Colitis (UC) is an inflammatory bowel disease characterised by diarrheoa, abdominal pain and rectal bleeding. Pro-inflammatory mediators, such as TNF-α, are up-regulated in UC, suppression of which has been proven to effectively reduce mucosal damage, colonic infiltration of macrophages and neutrophils and UC-associated tumour growth [192] (refer to Figure 1). As an idiopathic disease, the precise aetiology is unknown however the role of commensal bacteria and dysbiosis has emerged as potential causal factors [193]. Current treatments for IBD include anti-inflammatory drugs, dietary changes and surgery, but with the suggestion of commensal bacteria playing a role in pathogenesis, the therapeutic use of probiotics is being explored. 

The rationale behind probiotic therapy lies in the ability to modulate immune response. As UC is a predominant Th2-driven pathology, skewing of the immune response to a more tolerogenic tissue environment may alleviate the damaging effects of a dysregulated inflammatory response. Current therapies do aim to dampen-down these inflammatory responses, however patient responsiveness is variable and surgical procedures invasive; therefore probiotics pose an alternative, non-invasive therapeutic option. Of the studies conducted in UC mouse models, a number of *Lactobacillus* strains have been identified to modulate pro-inflammatory responses. Both *L. plantarum* 299v in IL-10 knockout mice [168] and *L. rhamnosus* GG in transgenic mice [169] have been shown to decrease levels of pro-inflammatory cytokines including IL-1β, TNF-α, IFN-γ and IL-12. Furthermore, levels of the anti-inflammatory cytokine IL-10, was augmented upon administration of *L. rhamnosus* GG [169]. In addition to direct immunomodulation, *Lactobacillus* alters epithelial barrier function: *L. rhamnosus* GG was found to decrease adherence of pathogenic *B. vulgatas* whereas the probiotic mix VSL#3 decreased expression of tight junction proteins, occludin, zonulin and claudin and prevented epithelial cell apoptosis in BALB/c mice [172]. VSL#3 has also been shown to reshape bacterial composition through enhancing species richness and diversity index [174]. In clinical trials, these findings have been related to gross pathological changes, with evidence of decreased tissue inflammation and enhanced disease remission and clinical responsiveness [173,194]. These studies clearly demonstrate the ability of probiotics to modulate the immune response in UC, skewing a predominant pro-inflammatory setting towards a more tolerogenic environment, characterised by down-regulation of pro-inflammatory cytokines such as TNFα, IL-1β and IFNγ and an up-regulation of anti-inflammatory cytokine IL-10. These effects appear to confer a health benefit, with evidence of enhanced epithelial barrier function, decreased tissue inflammation and a decrease in gross pathology scores (refer to Table 3). Furthermore, bacterial composition variance is emerging as an important factor in overall gut health and the development of gut-associated immune pathologies, with some evidence suggesting habitual consumption of health-promoting bacterial species can alter microbial populations in the gut, preventing UC. 

The health benefit of probiotic co-administration with prebiotics has also been explored in the context of *B. longum*, which was found to reduce levels of β-defensins, TNF-α, IL-1α and c-reactive protein (CRP) in a synbiotic “mix” [170]. In support, a more recent study conducted on UC patients, compared the effects of synbiotics with single probiotic treatment, finding only synbiotic therapy, to significantly suppress CRP levels [171]. In addition, CRP has been found to correlate with disease severity, implying potential use of CRP levels as a marker of UC progression [195]. Therefore if synbiotics act to reduce levels of these pathogenic factors, to an extent greater than probiotics alone, further studies must be conducted to explore optimal combinations of probiotics and prebiotics for therapeutic use.

#### 5.2.1. Anti-Tumoural Responses and NK Cell Activation

Natural killer cells (NKs) play a crucial role in tumour surveillance and anti-viral responses. NKs are directly activated by missing or altered self (MHC I), thus responding to suppressive, survival mechanisms elicited by viruses and tumour cells. In addition, NKs are indirectly activated by DCs which secrete soluble factors, such as IL-12, IL-18 and type I interferons. Priming of NKs subsequently leads to killing of tumour cells and the secretion of IFNγ and TNF-α, both pro-inflammatory cytokines capable of inducing cell-mediated immunity and further activation of antigen-presenting cells (DCs and macrophages) [196] (refer to Figure 1). Interestingly, recent studies have highlighted a role for probiotics in modulating the DC-NK interaction and subsequent anti-tumour immune responses. 

Probiotic *Lactobacillus* strains induce human PBMC secretion of pro-inflammatory cytokines, IL-12 and TNFα [121]. This has since been identified to be a DC-NK mediated immune response. Probiotic *Lactobacillus casei* Shirota (LcS) induces IL-12 and TNFα production which positively correlated to NK activity [125,129]. DC secretion of IL-12 primes NK activation and the subsequent secretion of TNF-α, therefore LcS may indirectly activate NK effector function via DCs. This potential probiotic mechanism was further proven by studies finding *Lactobacillus* strains induced APC production of IL-12, leading to NK activation and NK-derived IFNγ secretion, which not only has implications to innate responses but also CMI-mediated anti-tumour responses [114,132]. *Lactobacillus*-induced IL-12 however, was found to be abrogated in favour of enhanced IL-10 production, (favouring an immune-suppressive, pro-tumoral environment), by the probiotic, *Bifidobacterium*, suggesting combinatory probiotic therapy may be counter-productive to immunomodulation, in the context of NK-mediated immunity [132]. This further highlights a generalised distinction between probiotic strains, suggesting *Lactobacillus* predominantly induces a cytotoxic innate immune response (anti-tumour responses) whereas *Bifidobacterium* play a more regulatory role that may involve priming of regulatory T-cells (pro-tumoural responses). 

#### 5.2.2. Colorectal Cancer

Since the early work of Rudolph Virkchow in the 1800s, evidence has been accumulated supporting the hypothesis that chronic inflammation is important in cancer development. As inflammation contributes largely to the development and progression of ulcerative colitis (UC), mechanistic links have been made between ulcerative colitis and the development of colorectal cancer (CRC). Up-regulation of pro-inflammatory cytokines has been characterised in both UC and CRC [197] and development of CRC occurs within the same tissue site as UC; the chronic inflammation and subsequent tissue damage observed in UC may therefore be causing CRC. As probiotics and synbiotics have both been identified as potentially therapeutic, a similar therapeutic approach could be used for CRC. 

Although precise immunomodulatory mechanisms are yet to be clearly defined for probiotics in the context of CRC, probiotics have been found to modulate some pro-inflammatory molecules. A recent study highlighted *L. lactis* as capable of decreasing levels of hydrogen peroxide (H_2_O_2_) and enhancing catalase activity, resulting in decreased colonic damage and tissue inflammation, in a BALB/c mouse model [177]. Reactive oxygen species (ROS), such as hydrogen peroxide, are known to contribute to carcinogenesis and metastasis as part of the pro-inflammatory immune response, thus suggesting probiotics are able to target other inflammatory mediators as well as cytokines. Furthermore, *L. acidophilus* induced a decrease in mRNA expression of stromal-derived factor-1 receptor, CXCR4, suggesting a role in cancer metastasis prevention; on the down side however, also suppressed MHC-class I expression [180]. As antigen presenting cells (APCs) use MHC-I molecules to present antigen to CD8^+^ Tc, an important component of tumour surveillance, the findings of Chen *et al.*, present a potential drawback to the use of *L. acidophilus*. Activation of CD8^+^ Tc is desirable within the tumour microenvironment due to their anti-tumoural, cytotoxic characteristics; therefore, probiotic-mediated down-regulation of CD8^+^ Tc activation may perpetuate tumour growth and survival. In terms of gross pathology, *L. acidophilus* has been found to decrease tumour growth by 50% in comparison to non-treated mice and enhance the apoptosis of CT-26 cancer cells [180], suggesting the immunomodulatory effects of probiotic administration does have an impact on tissue ultrastructure. 

In addition to inhibiting pro-inflammatory mediators, the *Lactobacillus* mix VSL#3 was found to increase angiostatin, the endogenous inhibitor of angiogenesis and regulatory T-cells, in DSS-induced CRC mice [179]. In contrast, the study also observed an increase in the number of memory CD4^+^ T-cells and pro-inflammatory cytokines, IL-17 and TNFα. As cancer is a complex disease with evidence suggesting both pro- and anti-inflammatory mechanisms contributing to cancer progression, the varied immunomodulatory roles of probiotics suggests precise strain selection is required for accurate targeting of immune function in different pathologies including colorectal cancer. The stage of development of colorectal cancer may be the determining factor as to which probiotic strain is used. 

As diet is an important risk factor in susceptibility to CRC, with vitamin D_3_ and retinoic acid inferring positive effects [198] and heterocyclic amines in red meat acting as mutagens [199,200]; modulation of the dietary composites within the gastrointestinal tract, suggests scope for intervention with prebiotic substrates. As prebiotics are also known to work synergistically with probiotics to elicit beneficial effects on commensal populations and overall gut health, synbiotics have been explored as potential therapeutic agents in CRC. Studies using bifidobacteria and *Lactobacillus* strains in conjunction with prebiotics, such as inulin and oligofructose, have been found to off-set carcinogenesis. Evidence demonstrating inhibition of aberrant crypt formation, reduction of caecal pH [175] and increased short chain fatty acid (SCFA) production [176], suggests probiotics could be acting through a range of mechanisms. Direct immune modulation has also been found with *B. lactis* and *L. rhamnosus* which were shown to decrease IL-2 and inducible NO synthase, the enzyme responsible for nitric oxide production [176,178]. 

Collectively these studies highlight an important role for probiotics in modulation of pro-inflammatory and pro-tumoural immune responses (refer to Table 3). As colorectal cancer can arise from untreated colitis, these studies suggest potential use of probiotics as an anti-inflammatory therapeutic, utilised not only in pre-established colorectal cancer cases, but also as a preventative measure in patients exhibiting symptomatic signs of colitis or early-stage colorectal cancer. Furthermore, some experimental evidence suggests an important role for prebiotics, highlighting possible functional dependency of probiotics on prebiotic supplementation. 

### 5.3. Immunopathology: Hypersensitivity

#### 5.3.1. Allergy & Type I Hypersensitivity

Dysbiosis in the human gut is not only seen in gastrointestinal tract pathology but is also associated with disease at distal sites such as the airways of the lungs [201] and the skin [202,203]. Cross-talk between the microbiota and immune cells located in the mucosa and lamina propria not only primes and tolerises cells locally, but may initiate migration away from the gut towards the mesenteric lymph nodes and other lymphoid tissues inducing systemic immunomodulatory effects [204].

Hypersensitivity immune responses are undesirable over-exuberant immune responses mounted to an allergic antigen, or allergen. Hypersensitivity responses are categorised in accordance to the time taken for an immune response to be mounted and the effector cells and mediators involved. Type-1 hypersensitivity involves an anaphylactic response rapidly initiated to allergenic exposure. This response is predominated by mast cells and basophils, both granulocytes capable of secreting vasoactive amines, such as histamines, upon activation of its high-affinity Fc receptors by IgE (refer to Figure 1). This type of hypersensitivity also involves Th2 cell-derived mediators such as IL-4, IL-5 and IL-13, which induce immunoglobulin class switching to IgE. Modulation of this response, via T-cell subset skewing (e.g., Th2 to Th1 or enhancement of Treg), is a desirable therapeutic approach to allergic pathologies, such as atopic dermatitis and food hypersensitivity, modulatable by probiotic administration. 

A number of clinical studies examining differences in microflora between children who displayed early symptoms of atopy and healthy controls, showed a higher prevalence of *Clostridia* and *Bacteroides* but lower comparative numbers of *Lactobacilli* and bifidobacteria [205,206]. Although lactic acid bacteria have been shown to attenuate atopic dermatitis development in mice in a strain dependent manner [207,208], there appears to be little significant effect on human infants already displaying symptoms [209]. Further investigation in animal studies [210,211] and human clinical trials [212,213] has suggested that there may be a window of oportunity in early life before weaning in which probiotic therapy is of use in prevention. This positive probiotic effect arises as a consequence of immune education to microbes and the establishment of a more balanced immune system in the context of Th1, Th17 and Th2 responses.

Current treatments for allergic diseases such as asthma and food allergy are limited to management of the conditions, rather than cure. Probiotic immunomodulation is an attractive strategy with which to counterbalance Th2-skewed immune responses associated with allergy. *In vitro* studies using human peripheral blood mononuclear cells (PBMCs) from allergic patients have shown reduced expression of Th2-associated cytokines (IL-4 and IL-5) on stimulation with total extract of *Dermatophagoides pteronyssinus* (house dust mite) and prior treatment with lactic acid bacteria strains such as *Lactobacillus plantarum* [122]. Both *Lactococcus lactis* and *Lactobacillus plantarum*, induce high levels of IL-12 and IFNγ, suppressing Th2 differentiation [128,181]. In contrast, suppression of contact dermatitis in mice has been shown to be mediated by a *Lactobacillus acidophilus* L92-dependent generation of Tregs [214]. This effect is thought to result from strain-dependent tolerisation of DCs, increasing suppressor activity of natural Tregs as well as inducing Foxp3^+^ conversion through high expression of IL-10, TGFβ, COX-2 and indoleamine 2,3-dioxygenase [215,216].

Several studies, focussing on probiotic administration in type-1 hypersensitivity responses, revealed inhibitory characteristics for both *Lactobacillus* and *Bifidobacterium* strains. Mouse models have demonstrated the ability of *L. casei* and *B. longum* to inhibit IgE production [217,218,219]. Inhibition of antibody production prevents binding to Fc receptors on mast cells, thus inhibiting the secretion of vasoactive amines, such as histamines, and other inflammatory mediators, such as TNF-α. Indeed, histamine content was found to be decreased in *Bifidobacterium* treated rats [220] and TNF-α production was found to be inhibited by another *Lactobacillus* strain, *L. reuteri* [144], further supporting the role of probiotics in inhibiting mast cell effector function. Systemically, the inhibition of mast cell effector function by probiotics has been associated with preventable anaphylaxis [217]. The inhibition of IgE production is thought to be a consequence of direct action by probiotics on Th2 cells or APCs, which prime B-cell activation and class-switching. A large body of evidence demonstrates a role for *Lactobacillus* and *Bifidobacterium* strains in decreasing the levels of secreted IL-4 and IL-5. Both cytokines are Th2-derived, with IL-4 acting on B-cells to induce class-switching and on mast cells to induce degranulation and further cytokine production, and IL-5 inducing eosinophil degranulation. Specific strains found to inhibit IL-4 and IL-5 production include *L. casei* [219], *L. rhamnosus* [128], *B. longum* [218] and *B. infantis* [220]; see Table 1.

The effects of probiotics on type-I hypersensitivity responses have been further explored by a few studies, in terms of modulation of receptor expression and associated intracellular signalling cascades. Dev *et al.* [220] found the probiotic mix (LacB) composed of *B. longum* and *B. infantis*, inhibited the increase in histamine receptor-1 and histidine decarboxylase (HDC) expression and activity. Furthermore, histamine-2, known to inhibit hypersensitivity responses through activation of cAMP and increased intracellular calcium levels, was augmented by *L. reuteri* [144]. In addition, *L. casei* treatment in mice inhibited IgE production by inhibition of Syk/Lyn and MAPK signalling [219]. This provides some insight into probiotic targeting; suggesting probiotics mediate their anti-hypersensitivity effects through direct modulation of APC intracellular signalling with knock-on effects to down-stream effector cell priming. Unlike other probiotic immunomodulatory effects, there seems to be a commonality across probiotic strains in relation to its impact on hypersensitivity responses; whether the strain is *Lactobacillus* or *Bifidobacterium* derived, the immune response is shifted from a predominant Th2 response. Probiotics therefore may have a useful application in Th2-dominant pathologies whereby unnecessary immune responses are mounted against self or non-pathogenic antigens, such as atopic dermatitis or allergy.

Discrepancies have been seen in experiments testing the effectiveness of synbiotic mixtures. *Bifidobacterium breve* and oligosaccharide treatment used in a cohort of 29 patients with asthma, found a decrease in IL-5, IL-4 and IL-13 [182], yet the same synbiotic mix tested in atopic dermatitis infants found no significant difference in IL-5 levels. Furthermore, in response to allergen-specific stimuli, decreases in IL-12 production were found in conjunction with unchanging levels of Tregs [183]. Previously, an extensive study in a cohort of 90 infants, receiving the same synbiotic treatment, showed an increase in the number of faecal bifidobacteria and a decrease in the number of *Clostridium* colonies accompanied by a decrease in faecal pH; no significant changes were found in levels of IgE or the SCFA, acetic acid [184]. This study however did permit the use of topical corticosteroids during the experiment; a confounding variable that could have altered the effects of synbiotics on immune modulation. Thus, probiotics and synbiotics represent an efficacious approach to the control of hypersensitivity/allergy (refer to Table 3). There is a need to characterise immune hypersensitivity reactions and their modulation by these pro- and syn-biotics, as future use of these in the treatment of allergy will require an appreciation for strain variations in determining immune suppression or immune-deviation. The requirement for which will vary according to specific allergens and hypersensitivity.

#### 5.3.2. Coeliac Disease

At the other extreme of allergic responses is delayed type hypersensitivity (DTH) which includes the condition Coeliac disease, although this pathology encompasses many aspects of hypersensitivity reactions which culminate in an autoimmune-like disorder. This disease is associated with the gut mucosa triggered by an inappropriate immune response to the dietary antigen gluten (α-gliadin), more specifically toxic peptide elements resistant to proteolytic breakdown, in individuals who express the (HLA-DQ2/DQ8) antigen presenting molecule [187,221]. In this way, gliadin-derived peptides are presented to naïve and Th1 cells inducing secretion of IFNγ; additionally, gliadin-derived peptides (α-gliadin p31-43/49) induce high-level expression of membrane-bound IL-15 on intestinal epithelial cells inducing infiltration of intraepithelial lymphocytes [222,223,224]. IL-15 induces the expression of MICA by physiologically stressed intestinal epithelial cells, promoting the infiltration of NK cells and intraepithelial lymphocytes expressing the NKG2D receptor, resulting in the lysis of epithelial cells and the breakdown of the mucosal barrier, hence villous atrophy characteristic of coeliac disease [224,225,226,227] (refer to Figure 1). It has been shown that high levels of TNFα and IL-18, with surprisingly little IL-12, exist in people with this Th1-biased condition; IL18 drives the transcription factor T bet and thus induces naïve T helper cell differentiation towards Th1 [228]. The mechanisms underpinning this complex hypersensitivity type IV-predominated pathology are further complexed by the observations that antibodies play a role in this pathology; sIgA facilitates trancytosis of gliadin peptides across the epithelial barrier and autoantibodies (IgA and IgG) recognising tissue transglutaminase, normally involved in the breakdown of gliadin at the epithelial apical surface [229,230,231].

Coeliac patients have a dysbiosis in their gut microflora with lower variation in beneficial commensal strains of *Bifidobacterium* and the atypical expression of *Leuconostoc mesenteroides* and *L. carnosum* along with *Lactobacllius curvatus*, believed to be characteristic of coeliac patients [232,233]. The strain *Bifidobacterium longum* was significantly lower in coeliac patients than controls, whereas *B. bifidum* was higher in those with active disease [233]. Thus, a recent study looked at the effects of various bacterial strains isolated from coeliac patients on co-cultures of Caco-2 cells and PBMCs in the presence of the antigen gliadin and cytokine trigger IFNγ, then examined for cytokine release [187]. The bacterium *Shigella CBD8* was found to induce higher secretion of IL-12 and IFNγ in the presence of gliadin than stimulation with antigen alone, whereas stimulation with the strains *Bifidobacterium bifidum* induced higher levels of TNF-α than the other bacterial strains [187]. Thus strains present in the coeliac gut could either reduce disease risk or enhance the proinflammatory response to gliadin, and that the high levels of gram negative bacteria with fewer bifidobacteria than healthy individuals could be partially responsible for the disease pathogenesis [187]. To this end, the live strains of *Bifidobacterium lactis* and *Lactobacillus fermentum* were co-cultured with Caco-2 cells exposed to gliadin to monitor changes in transepithelial resistance; results demonstrated that *B. lactis* counteracted the effects of gliadin on TER, whereas *L. fermentum* had no effect at preventing increased permeability [186]. A recent review by Silano *et al.*, [234] discusses the fact that there appears to be a similar timing mechanism involved in the development of coeliac disease as is seen for nut allergy. There would appear to be a time-period in infancy where the risks associated with gluten intolerance can be minimised during weaning, the risk is increased before 3 months of age and after 7 months of age, it is also known that breast-feeding during the weaning period onto gluten halves the risk of developing coeliac disease.

Due to the variable immunological nature of coeliac disease, the use of probiotics is rather complex and associated impacts are hard to monitor experimentally (refer to Table 3). Studies using gliadin-immunised human HLA-DQ8 transgenic mice, found that two different strains of probiotic *Lactobacillus paracasei* and *Lactobacillus fermentum* increased the levels of antigen-specific TNF-α, and *Lactobacillus casei* induced a strong increase in CD4^+^ T cell population and IFNγ secretion [185,188]. However, it was later discovered that the administration of this *L.casei* in a murine NSAID-induced intestinal injury model, again expressing the HLA-DQ8 transgene, in fact had a preventative effect on villous atrophy, yet this effect was only mediated with prolonged use of the probiotic [235]. The literature available regarding the use of prebiotics as a therapy in this pathology is scarce. The accepted treatment for coeliac disease however, involves avoidance of dietary gluten and the use of inulin in gluten-free bread is being introduced as a method for producing wheat replacement foods with an improved consistency in order to help individuals adhere to the strict dietary regime [236]. Such use of the prebiotic, inulin, is likely to have an effect on the gut microbiota, potentially redressing the dysbiosis observed with this hypersensitivity-associated pathology.

## 6. Future Perspectives

This review has gone some way in highlighting the complexity of mechanistic effects elicited by the use of probiotics in the modulation of the human immune system. In the context of homeostatic gut mucosa and microbiota, the use of probiotics and indeed combination with prebiotics acts as a regimen for long health. In the case of gut pathology, whether inflammatory bowel disease, colorectal cancer or hypersensitivity, the use of prebiotics and probiotics in combination can have dramatic effects on the immune system, hence immunopathology. Future developments will only surface upon more extensive research into the mechanism underpinning these immunopathologies and how they may be modulated by prebiotics, probiotics and synbiotics. There are many areas of interest that can be mentioned; some which may be considered however, include (i) the role of pre-/probiotic modulators in immunosenescence, (ii) modulation of APC function, in particular mucosal macrophages, (iii) probiotic bacterial products (cell wall-associated and secreted) and finally, (iv) faecal transplantation.

Immunoscenescence: it is well established that the immune system deteriorates with old age. This includes thymic atrophy, hence reduction in overall T cell responses and a reduction in numbers and activity of NK cells [237,238]. Both these compartments of the immune system are vital to tumour surveillance; a reduction in which increases the prevalence of cancer in old age. Studies are surfacing which are focussing in on the ability of probiotics to augment the activity of DCs, NK cells and CD8^+^ Tc with some impressive results; responses not otherwise observed in healthy, younger populations [129,239].

Mucosal macrophages: represent an important APC, which remain resident in the responding tissue, as such are able to directly modulate local T cell, B cell, NK, DC, IEL and mast cell responses. There is an established literature for macrophage effector subsets, where type I macrophages are pro-inflammatory and type II are anti-inflammatory/regulatory. Manipulation of macrophage plasticity through probiotics, their secreted products and prebiotics, hold potential for harnessing their differential effector responses in the treatment of gut mucosal inflammatory bowel diseases, gut cancer and hypersensitivity (reviewed in [138]).

Probiotic bacterial products: probiotic bacteria may exert their immune-modulatory activity either by direct interaction of the bacterial cell with host immune cells or via secretable products. With the vast proliferation of the pattern recognition receptor literature, interest is growing with respect to characterising the commensal-associated molecular patterns (CAMPs). These patterns are already being described for their immunomodulatory functionality and include cell wall components such as *Lactobacillus plantarum* lipoteichoic acid, which exhibits anti-inflammatory effects [240,241] and C-type lectins, which interact with DC-SIGN on DCs, resulting in the induction of Tregs [115]. Finally, the characterisation of the lactobacillus, exoproteosome, is likely to introduce many secretable immunomodulatory molecules (reviewed in [242]).

Faecal transplantation: It is generally considered that the GIT microbiota of healthy adults remains in a stable state of eubiosis. However as work continues on probiotic research, it is increasingly apparent that factors such as mode of delivery and feeding regimen in early life in conjunction with host genetics, antibiotic usage and life style, influence the composition of the microbiota making it genetically and metabolically unique to the individual [243]. The extent of this variation is likely to be the primary source of inconclusive results from clinical trials testing prebiotic/probiotic/symbiotic formulations in humans, as one person’s microbial balance may not suit another. Yatsunenko *et al.*, [244], revealed that GIT microbial composition is most alike between cohabiting individuals compared to those from other families. This similarity may provide a unique resource for rebooting the GIT microbiota resulting in alleviation of disease associated with microbial dysbiosis. Allogenic faecal transplantation has been successfully used in the resolution of *C. difficile* infections [245] and presents promise for the management of symptoms in IBD patients [245]. Furthermore, faecal transplantation may provide a strategy to prevent or treat inappropriate immune responses at sites distal to the GIT, such as those seen in asthma and allergic rhinitis, through Th1-mediated cross-regulation of Th2 responses and generation of Tregs, which may suppress effector T cells mobilised in allergy. 

## 7. Conclusions

In addition to playing an important role in barrier defence, probiotics have an ever-unfolding role to play in the modulation of mucosal immunity. Probiotic bacteria can modulate the activity of many cells of the immune system, including innate system NKs, DCs, macrophages, epithelial cells and granulocytes, as well as adaptive system Th1, Th2, Th17, Treg, Tc and B cells. Thus, probiotic bacteria have the potential to modulate any part of the immune system in the context of acute responses to intracellular or extracellular pathogens or chronic responses observed in dysregulated immunopathological conditions such as IBD, colorectal cancer and hypersensitivity. Modulation of immunopathology to the advantage of the human host can only realistically occur upon a full knowledge of the modulatory capabilities of the probiotic and a full appreciation of the mechanisms underpinning the pathology. Probiotic bacterial strains can be generalised to exert immune-activation, -deviation or -regulation/suppression responses. Thus, selection of probiotic strains, and indeed combinations of probiotics, will be formulated upon careful consideration of the disease mechanisms and the desired immunomodulatory effect. Finally, the growing body of evidence for the suitability of prebiotics in immunomodulation and formulation with probiotic strains, as synbiotics, represents a realistic approach in disease modification. The probiotic species acting as a quick-fix immunomodulatory “plaster” and the prebiotic facilitating commensal organisms, modulating the dysbiosis in the gut microbiota and further modifying the immune system to our benefit—healing from within!!
